# Prevalence of High Resilience in Old Age and Association with Perceived Threat of COVID-19—Results from a Representative Survey

**DOI:** 10.3390/ijerph18137173

**Published:** 2021-07-05

**Authors:** Elena Caroline Weitzel, Margrit Löbner, Susanne Röhr, Alexander Pabst, Ulrich Reininghaus, Steffi G. Riedel-Heller

**Affiliations:** 1 Institute of Social Medicine, Occupational Health and Public Health (ISAP), Medical Faculty, University of Leipzig, Philipp-Rosenthal-Str. 55, 04103 Leipzig, Germany; Margrit.Loebner@medizin.uni-leipzig.de (M.L.); Susanne.Roehr@medizin.uni-leipzig.de (S.R.); Alexander.Pabst@medizin.uni-leipzig.de (A.P.); Steffi.Riedel-Heller@medizin.uni-leipzig.de (S.G.R.-H.); 2 Global Brain Health Institute (GBHI), Trinity College Dublin, D02 PN40 Dublin, Ireland; 3 Department of Public Mental Health, Central Institute of Mental Health, Medical Faculty Mannheim, Heidelberg University, 68159 Mannheim, Germany; Ulrich.Reininghaus@zi-mannheim.de; 4 Centre for Epidemiology and Public Health, Health Service and Population Research Department, Institute of Psychiatry, Psychology & Neuroscience, King’s College London, London WC2R 2LS, UK; 5 ESRC Centre for Society and Mental Health, King’s College London, London WC2R 2LS, UK

**Keywords:** high resilience, old age, COVID-19

## Abstract

Little is known about resilience in old age and its manifestation during the COVID-19 pandemic. This study aims to estimate the prevalence of high resilience in the German old age population. We further examine the socio-demographic correlates and whether high resilience reflects on older adults’ perception of the threat posed by COVID-19. The data were derived from a representative telephone survey of n = 1005 older adults (≥65 years) during the first COVID-19 lockdown. Assessments included socio-demographic variables, the perceived threat of COVID-19, and high resilience (Brief Resilience Scale; cutoff: ≥4.31). The association between high resilience and threat from COVID-19 was analyzed using ordinal logistic regression. The study sample had a mean age (*SD*) of 75.5 (7.1) years, and n = 566 (56.3%) were female. The estimated prevalence of high resilience was 18.7% (95% CI = [16.3; 21.2]). High resilience was more prevalent in the younger age group and participants with higher education levels. High resilience was significantly associated with a lower perception of threat from COVID-19. The results of the representative survey in the German old age population showed that one out of five adults aged 65 years and older had high resilience. Older adults with high resilience tended to feel less threatened by COVID-19. Further research on resilience in old age is needed to support vulnerable groups in the context of care.

## 1. Introduction

Resilience is defined as a positive adaption to negative life circumstances [1] and con-sists of a conglomerate of functional behaviors and thoughts [2]. The key characteristics of high resilience in old age include adaptive coping styles, optimism, and positive emotions [3]. Social support is also an essential resilience resource [3,4,5,6,7]. The nature of resilience has been extensively discussed [1]. Resilience has partly been considered a personality trait [7,8]. Now, there is growing consensus that resilience is more adequately understood as a learnable process, which develops dynamically and can be trained [1,2,9,10]. Resilience is more strongly pronounced in older individuals, who have had broader life experience and presumably have mastered previous crises [11]. Taking a closer look at high resilience during old age can reveal important insights into the pathways to successful aging.

Until now, the prevalence of high resilience has not been estimated in the old age population. In line with Röhr et al. (2020), we use the term “old age population” to define the population of individuals aged 65 years and older. Studies on socio-demographic correlates of resilience among the old are also scarce and often refer to vulnerable subgroups with limited generalizability. The few findings are heterogeneous with regard to resilience patterns considering the effects of age [5,8,12], gender [5,7,8,13], or education [4,5,7,8]. The scarcity of the literature and the contradictory findings of previous research emphasize the need for representative studies examining the prevalence of high resilience and its socio-demographic correlates in the old age population.

There is a need to define how high resilience in old age can be assessed. Previous studies have measured relevant outcome variables associated with resilience in old age, such as high life satisfaction or the absence of negative health outcomes [14], but not resilience itself. Various scales exist to assess resilience more directly [15,16,17,18,19]. The Brief Resilience Scale (BRS) aims to assess resilience in its original meaning as the “ability to bounce back or recover from stress” [16] (p. 194). This short self-report instrument has been broadly validated and the results can be classified into low, normal, and high resilience [20,21,22]. Therefore, the BRS represents a particularly well-suited instrument to assess the prevalence of high resilience in the old age population.

Recently, handling adverse life circumstances has gained tragic relevance. The on-going pandemic of coronavirus disease 19 (COVID-19) requires adaptation efforts and may lead to an increase in mental disorders in the long term [23]. Despite greater health threats for adults aged 65 years and older [24], preliminary findings indicate that the old age population handles the pandemic particularly well [25,26,27,28]. It is widely assumed that this can be attributed to the high resilience in this age group [28,29,30,31]. Preliminary studies have shown the positive effects of resilience on COVID-19-related anxiety and concerns [21,32,33]. Until now, how high resilience is reflected in COVID-19-related attitudes among the elderly has not been examined. The present study attempts to fill this research gap.

Synoptically, this study aims to investigate the prevalence of high resilience in the German old age population with a representative sample of individuals aged 65 years and older and to further identify associated factors. Gaining further knowledge about resilience patterns in the old age population can give important insights into successful aging and the relevance of resilience during the current pandemic. The following research questions are examined:

1. What is the prevalence rate of high resilience within a representative sample of the German old age population (65+)?
2. How are socio-demographic factors associated with high resilience in the old age population?
3. How is high resilience associated with the perceived threat of COVID-19 in the old age population?

## 2. Materials and Methods

The data came from a representative telephone survey of n = 1005 individuals aged 65 years and older in Germany during the COVID-19 lockdown in April 2020. The main results regarding the mental and social health outcomes of the lockdown have already been reported elsewhere [28].

### 2.1. Study Design and Sample

Computer-assisted telephone interviews were conducted by the social research institute USUMA from 6 to 25 April during the nationwide lockdown measures. The details of the sampling process have been comprehensively reported by Röhr et al. [28].

### 2.2. Assessments

#### 2.2.1. Socio-Demographic Characteristics

The socio-demographic variables included age, gender (female, male, or other), marital status (married, single, divorced, or widowed), educational qualification (highest school degree), and vocational qualification (highest occupational degree or training). Age was divided into three groups (65–74 years, youngest-old; 75–84 years, middle-old; >85 years, oldest-old) in order to best represent the old age population [34]. Due to a small number of individuals with high resilience among participants aged 85 years and older, we combined the participants aged 75 years and older into one age group. To balance the group sizes according to marital status, we included participants with a marital status other than married in one group (single, divorced, and widowed). Levels of education and vocational qualifications were classified into low, middle, and high education according to the Comparative Analysis of Social Mobility in Industrial Nations (CASMIN) classification [35].

#### 2.2.2. Perceived Threat of COVID-19

Participants were asked about their agreement with the statement “I feel personally threatened by COVID-19”, with response options ranging from “totally disagree” to “to-tally agree” on a 5-point Likert scale.

#### 2.2.3. High Resilience

Resilience was assessed with the validated German adaptation [36] of the Brief Resilience Scale (BRS) [16]. This comprises six items to be answered on a 5-point Likert-scale ranging from “totally disagree” to “totally agree”. Half of the items are reverse worded and coded. Participants were asked to indicate their agreement to the German version [36] of the following statements: “I tend to bounce back quickly after hard times”; “I have a hard time making it through stressful events”; “It does not take me long to recover from a stressful event”; “It is hard for me to snap back when something bad happens”; “I usually come through difficult times with little trouble”; “I tend to take a long time to get over set-backs in my life” [16]. We computed a mean score, with higher values indicating higher resilience (range: 1–5). Mean scores were divided into three categories (low resilience = 1.00–2.99, normal resilience = 3.00–4.30, and high resilience = 4.31–5.00) [16,20,21,22] in order to estimate the prevalence of high resilience. To identify factors particularly associated with high resilience, we combined participants with low and normal resilience into one group.

### 2.3. Data Analysis

We weighted the results in terms of gender, age, and region based on census data in order to ensure representativeness among these demographics. The weighted frequency of high resilience in the representative sample and subgroups was used to estimate the prevalence of high resilience in the German old age population. We used χ^2^ tests to compare participants with high versus low or normal resilience with regard to socio-demographic variables.

The association of high resilience and the perceived threat of COVID-19 was analyzed via ordinal regression analysis (OLR). OLR is a suitable method for measuring the association of independent categorical or continuous variables with an ordinal-scaled outcome variable [37]. The outcome variable was the perceived threat of COVID-19. The independent variables were resilience (high or normal/low) and the following covariates: age (>75 years or 65–74 years), gender (female or male), marital status (single/divorced/widowed or married), and education (low and middle or high). A significant β-coefficient indicated relevant differences between participants with high resilience compared to participants with low or normal resilience in the outcome variable. For better interpretability, we computed and reported odds ratios (OR) to indicate a change in the outcome variable due to a change in the independent variable. Analyses were performed with SPSS (Version 25, IBM Corp., Armonk, NY, USA). Significance was defined at α ≤ 0.05.

## 3. Results

Of the N = 1863 randomly selected subjects aged 65 years and older, n = 200 (10.7%) refused to participate in the survey and n = 658 (35.3%) could not be reached, resulting in a study sample of n = 1005. N = 566 (56.3%) were women and the mean age of the sample was 75.5 years (*SD* = 7.11). Participants reported low, medium, and high education in similar proportions (low: n = 279, 27.7%; middle: n = 352, 35.1%; high: n = 360, 35.9%).

### 3.1. Prevalence of Resilience

Information on resilience was available for n = 954 (94.9%) of the study sample. The estimated prevalence of high resilience was 18.7% (95% CI = [16.3; 21.2], n = 168). The prevalence of low or normal resilience was 81.3% (95% CI = [78.8; 83.7], n = 768). Table 1 shows the estimated prevalence rates of high and normal or low resilience in the German old age population with regard to socio-demographic characteristics.

### 3.2. High Resilience and Socio-Demographic Characteristics

High resilience differed significantly with regard to the different age groups among the old (χ^2^(1) = 9.87, *p* = 0.002). The prevalence of high resilience was higher in participants aged 65-74 years (22.7%, n = 107) than in those aged 75 years and older (14.8%, n = 79).

The prevalence of high resilience in the old did not differ significantly according to gender (χ^2^(1) = 2.96, *p* = 0.085). High resilience was prevalent in 21.1% (n = 90) of older men and in 16.7% (n = 96) of older women.

The prevalence of high resilience did not differ between married older adults (19.5%, n = 86) and older adults who were divorced, single, or widowed (17.7%, n = 99; χ^2^(1) = 0.48, *p* = 0.486).

High resilience varied significantly with regard to education (χ^2^(2) = 13.61, p = 0.001). The prevalence of high resilience was equally high in those with high education (21.8%, n = 80) and those with middle education (21.4%, n = 73). A lower prevalence of high resilience was found in older adults with low education (11.2%, n = 32).

### 3.3. Resilience and the Perceived Threat of COVID-19

**Figure 1:**
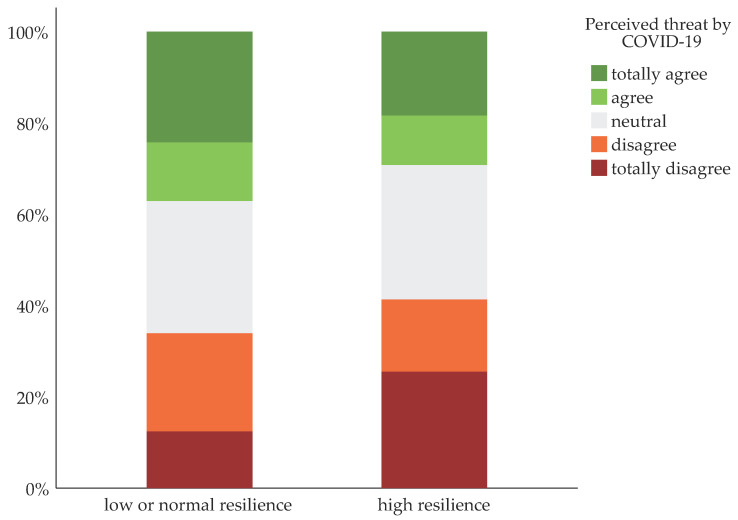
The perceived threat of COVID-19 in the German old age population with regard to high resilience and low or normal resilience (representative sample of n = 1005; age ≥ 65 years).

The frequencies of the perceived threat of COVID-19 in reference to resilience are listed in Table 2. The perceived threat of COVID-19 varied significantly between old adults with high resilience and old adults with low or normal resilience (χ^2^(4) = 21.37, *p* < 0.001). In total, 25.5% (n = 46) of older adults with high resilience strongly disagreed when asked if they felt threatened by COVID-19, compared to 12.4% (n = 97) of those with low or normal resilience. Among older adults with high resilience, 18.4% (n = 34) strongly agreed when asked if they felt threatened by COVID-19, whereas 24.3% (n = 180) of those with low or normal resilience strongly agreed. Response behavior with regard to high and low or normal resilience is displayed in Figure 1.

The results of the ordinal logistic regression analysis are listed in Table 3. The final model predicted the perceived threat of COVID-19 significantly better than the intercept-only model (*p* = 0.031), although the explained variance was small (Nagelkerke’s pseudo R^2^ = 0.015). The association between resilience and the perceived threat of COVID-19 was significant. High resilience significantly predicted lower perceived threat from COVID-19 among the old (OR = 0.657, *p* = 0.005).

## 4. Discussion

To our knowledge, this was the first study to determine the prevalence of high resilience in a representative sample of the German old age population aged 65 years and older. High resilience was prevalent in about one fifth (18.7%) of individuals aged 65 years and above. Our results indicated that it was common for the German population aged 65 and older to adapt well to challenging conditions and crises. Our results stand out from studies examining high resilience in younger people. For instance, a study by Whatnall et al. (2019) on university students found that only about one out of ten students had high resilience [20]. This emphasizes once again that resilience is a dynamically developing process formed by life experience and the successful mastery of previous crises [1,2], resulting in more pronounced resilience in older age groups. The course of resilience over the life span should be examined in longitudinal studies to verify this assertion with further focus on the underlying causes.

Among the old, the prevalence rates of high resilience differed according to age. The prevalence of high resilience was greater in individuals aged 65–74 years (22.7%) than in those aged 75 years and older (14.8%). Previous studies found heterogeneous results regarding the association between age and resilience among the old [5,7,8]. In accordance with Perna et al. (2012), our results suggest that after the age of 65, the prevalence of high resilience decreases with greater age. One possible explanation for this is the high frequency of social loss experiences among the oldest-old, which represent important resilience resources [3,4,5,6,7] and challenge psychological well-being [38]. Future studies should take the association between resilience and social loss experiences in old age into account.

The vast majority of previous studies did not find any gender differences in resilience among the old [7,8,13], although they had limited generalizability. Nygren et al. (2005) only included individuals aged 85 years and older. Perna et al. (2012) and Wells (2010) did not report prevalence rates for high resilience according to gender. In the study by Netuveli et al. (2008), higher resilience was associated with the female gender. This study was the first to estimate the gender-specific prevalence of high resilience in a comprehensive, representative sample of adults aged 65 years and older at the population level. In agreement with the vast majority of previous studies, we did not find gender differences in the prevalence of high resilience, although we observed a tendency for a higher prevalence in men (21.1% vs. 16.7%, *p* = 0.085). This contrast with findings from Netuveli et al. (2008) might be traced back to different methodological approaches. Netuveli et al. (2008) only included participants exposed to adversity during the study period and conceptualized resilience as the deterioration and amelioration of general health. Thus, it is possible that, although women recover better from negative health repercussions after adversity, this is not reflected in their confidence in overcoming crises [7,8,13]. The gender-specific facets of resilience comparing self-perception and health outcomes deserve further research attention.

We found significant differences in the prevalence of resilience with regard to education. Whereas high resilience was nearly equally distributed among participants with middle or high education (21.4% and 21.8%), only one out of ten participants (11.2%) with low education was found to be highly resilient. Previous research concerning education and resilience in old age shows heterogeneous results. Some studies did not find associations between education and resilience [5,7]. Others identified higher rates of high resilience in those with more education [4,8] and relevant health effects of education, particularly in old age [39]. In line with the latter, our findings emphasize that education lays the foundation for people to feel well-prepared to face crises during old age.

Presently, the COVID-19 pandemic is having broad, unpleasant psychosocial consequences [40]. During the first wave of the COVID-19 pandemic, older adults dealt particularly well with these challenging conditions [25,26,28]. The authors of previous studies assumed that stable well-being in the old during the first wave of the COVID-19 pandemic could be traced back to the high resilience in older age groups [28,29,30,31]. Our results support these assumptions: we found that older adults with high resilience tended to feel less threatened by COVID-19. Future studies should shed more light on the effects and mechanisms of high resilience during the ongoing pandemic. Learning more about high resilience in old age might provide an important starting point for interventions supporting more vulnerable subgroups in the old age population.

With regard to the COVID-19 pandemic, interventions should be aimed at specific subgroups of older adults (e.g., those with lower educational status or socioeconomic status, oldest-old individuals, etc.) to support resilience and mitigate the threat of COVID-19. To date, there is evidence of the efficacy of mindfulness-based, CBT-based, and mixed interventions [10]. Another starting point could be the initiation and maintenance of social support, which is particularly relevant for developing resilience [3,4,5,6,7]. How this can be implemented during the pandemic should be investigated in further studies. In the context of the pandemic, the relevance of digital services could be further promoted because they are widely available [41] and do not involve face-to-face contact.

### Limitations

Our results are based on a large, representative survey of the German old age population during the COVID-19 lockdown, and thus represent a cross-sectional depiction of resilience patterns in old age. In line with previous studies [32,33], and due to the unpredictable nature of the pandemic, we assessed the perceived threat of COVID-19 using a general measure. Which domains of life are perceived to be threatened by the pandemic remains unclear. To our knowledge, no validated instruments for assessing the perceived threat of COVID-19 in older adults existed at the time of the assessment (April 2020). Due to the little explained variance in our statistical model, the association between high resilience and the perceived threat of COVID-19 should be interpreted with caution, as further underlying variables are suggested to be present. Further longitudinal studies are necessary in order to examine the course of resilience in old age.

## 5. Conclusions

This study aimed to fill gaps in the research considering resilience in old age. In this large, representative sample of the German old age population, we found that high resilience was prevalent in about one fifth of adults aged 65 years and older. Younger age and higher education were identified as relevant socio-demographic correlates of resilience in old age. Our results provide initial indications that resilience is reflected in how we handle the threats of the COVID-19 pandemic. The determinants and effects of resilience in old age represent promising starting points to illuminate pathways to successful aging that deserve to be given further attention in future studies.

## Figures and Tables

**Table 1:** The prevalence of high resilience and socio-demographic correlates in the German old age population (representative sample of n = 1005; age ≥ 65 years).

	Total	Low or Normal Resilience			High Resilience			
	n	n	%	95% CI	n	%	95% CI	*p*
Total	954	768	81.3	[78.8; 83.3]	186	18.7	[16.3; 21.2]	
Age group in years	954							**0.002**
65-74	466	359	77.3	[73.3; 80.9]	107	22.7	[19.1; 26.7]	
≥ 75	488	409	85.2	[81.9; 88.1]	79	14.8	[11.9; 18.1]	
Gender	954							0.085
Male	466	325	78.9	[74.8; 82.6]	90	21.1	[17.4; 25.2]	
Female	488	443	83.3	[79.9; 86.2]	96	16.7	[13.8; 20.1]	
Marital status	950							0.486
Married	449	363	80.5	[77.0; 83.7]	86	19.5	[16.3; 23.0]	
Single/divorced/widowed	501	402	82.3	[78.4; 85.7]	99	17.7	[14.3; 21.6]	
Education	943							**0.001**
Low	273	241	88.8	[84.6; 82.2]	32	11.2	[7.8; 15.4]	
Middle	332	259	78.6	[73.7; 82.5]	73	21.4	[17.2; 26.0]	
High	338	258	78.2	[73.6; 82.3]	80	21.8	[17.7; 26.4]	

Notes. Significant differences between groups were assessed via Pearson’s χ^2^. n are an unweighted count of the study sample, and percentages are weighted by gender, age, and region. Bold: Significant differences between groups.

**Table 2:** High resilience and the perceived threat of COVID-19 in the German old age population aged 65 years and older (n = 1005).

	Total		Low or Normal Resilience		High Resilience		Group Difference
	n	%	n	%	n	%	*p*
Perceived threat of COVID-19							**<0.001**
Strongly disagree	143	14.8	97	12.4	46	25.5	
Disagree	197	20.5	163	21.5	34	15.8	
Neither agree nor disagree	277	29.0	221	28.9	56	29.4	
Agree	122	12.5	106	12.9	16	10.9	
Strongly Agree	214	23.2	180	24.3	34	18.4	

Notes. Data on the perceived threat of COVID-19 were available for n = 953 (94.8%) of the study sample. The significant group differences were assessed with Pearson’s χ^2^. n are an unweighted count of the study sample, and percentages are weighted by gender, age, and region.

**Table 3:** The association of socio-demographic factors with the perceived threat of COVID-19 in the German old age population (representative sample of n = 1005; age ≥ 65 years): results of the multivariable ordinal logistic regression analysis ^a^.

	Perceived threat of COVID-19		
	OR	95% CI	*p*
*Socio-demographic factors*			
Gender (ref. male)			
Female	0.978	[0.767; 1.246]	0.856
Age group in years (ref. 65–74)			
≥ 75	1.175	[0.931; 1.482]	0.175
Marital status (ref. married)			
Single/ divorced/ widowed	0.892	[0.700; 1.135]	0.353
Education (ref. high)			
Low	1.101	[0.823; 1.472]	0.517
Middle	0.876	[0.666; 1.151]	0.342
*Resilience*			
Resilience (ref. low/normal)			
high	0.657	[0.490; 0.883]	**0.005**

Notes. OR = odds ratio, CI = confidence interval, after casewise exclusion of missing data analysis was performed with n = 901 (89.65%) of study participants. ^a^ = regression was performed with the outcome variable perceived threat of COVID-19; independent variables were gender, age group, education, marital status, and resilience.

## Data Availability

The data presented in this study are available on request from the corresponding author. The data are not publicly available due to privacy.
